# A Dataset for Temporal Semantic Segmentation Dedicated to Smart Mobility of Wheelchairs on Sidewalks

**DOI:** 10.3390/jimaging8080216

**Published:** 2022-08-07

**Authors:** Benoit Decoux, Redouane Khemmar, Nicolas Ragot, Arthur Venon, Marcos Grassi-Pampuch, Antoine Mauri, Louis Lecrosnier, Vishnu Pradeep

**Affiliations:** Normandie University, Unirouen, Esigelec, Irseem, 76000 Rouen, France

**Keywords:** dataset, semantic segmentation, convolutional neural network, deep learning, smart mobility

## Abstract

In smart mobility, the semantic segmentation of images is an important task for a good understanding of the environment. In recent years, many studies have been made on this subject, in the field of Autonomous Vehicles on roads. Some image datasets are available for learning semantic segmentation models, leading to very good performance. However, for other types of autonomous mobile systems like Electric Wheelchairs (EW) on sidewalks, there is no specific dataset. Our contribution presented in this article is twofold: (1) the proposal of a new dataset of short sequences of exterior images of street scenes taken from viewpoints located on sidewalks, in a 3D virtual environment (CARLA); (2) a convolutional neural network (CNN) adapted for temporal processing and including additional techniques to improve its accuracy. Our dataset includes a smaller subset, made of image pairs taken from the same places in the maps of the virtual environment, but from different viewpoints: one located on the road and the other located on the sidewalk. This additional set is aimed at showing the importance of the viewpoint in the result of semantic segmentation.

## 1. Introduction

Robust scene perception is a principal requirement within many autonomous mobile applications. Accurate and efficient object detection techniques, depth estimation and semantic segmentation offer highly functional information about the operational environment. The present work focuses on semantic segmentation, which is the process of classifying image pixels into specific categories related to the environment. For training and testing data-driven models that perform this task, the availability of datasets of images with ground-truth annotations is crucial. Whatever the image-processing task involved, getting annotations is generally partly manual, and this operation becomes very tedious for semantic segmentation as well as for depth estimation, as they must be at the pixel level. There are a few datasets available for semantic segmentation, but most of them are dedicated to experiments on autonomous vehicles with a viewpoint located on the roads. In our case, we are interested in the autonomous mobility of an Electric Wheelchair for outdoor applications. Thus, the existing datasets are not well fitted to our problem, in which the viewpoint of images is located on sidewalks. Even if sidewalks are often visible in existing datasets, this difference of viewpoint may be a too important bias in the development of models designed specifically for sidewalks. Regarding semantic segmentation models, most of them use single images. However, temporal information which exists in image sequences can be very useful, as successive images of sequences are generally highly similar. Those considerations led us to develop our own dataset of image sequences, taken in an outdoor virtual environment from viewpoints located on sidewalks. Our contributions include a dataset of synthetic scenes generated using CARLA simulator [1] and a temporally distributed network based on DeepLabV3+ architecture [2]. The remainder of this paper is organized as follows. Section 2 presents the state of the art of existing datasets of images for semantic segmentation and models, with a focus on those dealing with image sequences. Section 3 describes our contributions and experiments. Section 4 presents results obtained with our dataset and model, and Section 5 concludes this work.

## 2. Related Work and Motivations

### 2.1. Datasets

Standard benchmarks for semantic video segmentation include Cityscapes [3] and Camvid [4] datasets. Cityscapes is available in 2 versions: isolated images and sequences. The image version includes 5000 of size 2048 × 1024 annotated for semantic segmentation, for 19 classes. Images are taken from urban environments, under varying weather conditions and in 50 different cities. Another part of the dataset includes 20,000 images coarsely annotated. The sequence version of the dataset includes not only the annotated images but also intermediate ones. Video snippets are made of 30 frames, taken at 17 FPS (Frames Per Second), from which the 20th one is annotated for semantic segmentation. This version of the dataset can be used by models dealing with the temporal aspect of the sequences. Camvid consists of 5 video sequences in which every frame is labeled, with a total number of annotated images of 701. NYUDv2 is another dataset that includes video sequences of various indoor scenes recorded with an RGB-Depth camera [5]. DriveSeg [6] is another recent dataset dedicated to Dynamic Driving Scene Segmentation. Other real-world datasets for semantic or instance segmentation include [7], dedicated to small overlooked indoor objects.

Another way to get annotated sequences of images is to use 3D virtual images generated by a software, like in GTA-5 [8] (24,966 images, 19 classes), SYNTHIA [9] (9400 images, 16 classes), Virtual Kitty [10], etc. However, when using virtual images during learning and real images during inference, a loss of accuracy called “domain shift” is stated. So there is a need to transpose the result from the original domain to the destination domain, by finding solutions to reduce this phenomenon. Standard benchmark adaptation tasks are for example SYNTHIA-to-Cityscapes and GTA-to-Cityscapes. The problem can be tackled in various ways like Generative Adversarial Networks (GAN) [11] or by learning the correlation between semantic and depth [12]. The main advantage of virtual datasets is that they can be easily generated, with various conditions and without technical constraints. Their main drawback is that their images are more or less realistic and then we need domain adaptation techniques to try to fill in the gap between the virtual and real domains.

In this work, we propose a dataset of virtual images generated through CARLA simulator [1], an open-source simulator for experiments on autonomous vehicles in which various maps of urban environments can be loaded. We have chosen this simulator as it allows us to displace the viewpoint of images from road to sidewalks. In the present study, we focus on learning and validating a model on the same virtual dataset, so we do not present experiments on domain adaptation.

### 2.2. Semantic Segmentation by Convolutional Neural Networks

#### 2.2.1. Fixed Image Models

The pioneering work on semantic segmentation using Convolutional Neural Networks (CNN) include various approaches like deconvolutional networks [13], hierarchical features [14], recurrent CNN [15], and zoom-out features [16]. A big step is done with models having an encoder-decoder structure, like LDN (Learning Deconvolution Network) [17], UNet [18], the FCN (Fully Convolutional Network) [19] and SegNet [20]. In those models, the decoder is used to recover the full resolution of the inputs. Other models use alternative solutions for the decoder part. PSPNet [21] performs spatial pyramid pooling to capture multi-scale information. DeepLab [22] uses “à trous” convolution to capture information of the images at different spatial scales (ASPP: Atrous Spatial Pyramid Pooling). In DeepLabV3+ [2], a decoder module is added after the ASPP step to refine the segmentation results, especially along object boundaries. Many other techniques have been proposed to increase the accuracy of these models. In Bayesian-SegNet [23], uncertainty is introduced in the result of the segmentation, allowing accuracy to be improved by 2 to 3%. In DenseASPP [24], the principle of ASPP is extended but with a more dense connection, leading to better accuracy. In [25], Gated Fully Fusion (GFF) selectively fuses features from multiple levels using gates in a fully connected way. Dual Attention Network (DANet) [26] captures feature dependencies in the spatial and channel dimensions. Object-Context Network (OCNet) [27] uses, for each pixel of the semantic map, object context to incorporate the information of objects belonging to the same category. PSANet [28] captures pixel-wise relation by a convolution layer and relative position information in the spatial dimension. EncNet [29] introduces a channel attention mechanism to capture global context. Attentional Class Feature Network [30] uses class information to integrate context. In Directed Acyclic Graph–Recurrent Neural Network (DAG-RNN) [31], contextual dependencies over image regions are captured by recurrent neurons. Another strategy to improve accuracy consists of Multi-Task Learning (MTL), for example by jointly learning the depth and semantic segmentation [32,33], or by using a two-stream CNN: a segmentation stream and semantic boundaries stream, the first one communicating with the second one in a gated way, like in [34].

#### 2.2.2. Image Sequence Models

The idea of temporal processing is to exploit the high redundancy that exists between successive images of a sequence, in order to improve accuracy. A natural way of processing temporal information in image sequences is to use some specific processing units or connection schemes, different from standard convolutional neurons. These units include Recurrent Neural Networks (RNN) [35], Long Short-Term Memory units (LSTM) [36,37] and Gated Recurrent Units (GRU) [38]. GRU has a simpler architecture than LSTM, but both have a high processing cost, partly due to the full connections to the previous layer. To overcome this problem, convolutional LSTM (ConvLSTM) [39] and convolutional GRU (ConvGRU) [40] have been proposed, in which full connections are replaced by convolutional ones. LSTM units can be integrated into any classical CNN like FCN [41], SegNet or ICNet [42].

The use of optical flow is another approach to integrating temporal information into semantic segmentation [43]. However, CNN architectures have to be adapted to limit the increase in processing cost. Some models exploit the observation that features can be reused in images to reduce computation. This principle is implemented in the form of a CNN with two branches: one made of a fully convolutional network, processing only keyframes, and the other processing all the images but with the propagation of the features from the first branch. In Deep Feature Flow [44], features are propagated from keyframes to the following ones. Another way of using video information to improve image semantic segmentation is video propagation and label relaxation [45]. After this learning, estimation is applied to isolated images. In [46], keyframes are selected on the basis of their difference from the previous frames, and the propagation takes place from the lowest convolutional layers to reduce the processing cost. In [47], keyframes are selected at regular intervals. and the features are propagated by a specific function, based on Deep Feature Flow [44]. The optical flow of adjacent frames can be used for warping internal network representations across time and can be combined with existing CNN architectures [48]. In DWNet (Dynamic Warping Network) [49], warping features are integrated into the learning, making it dynamic, and allowing an improvement in accuracy. In [50], semantic and optical flow are jointly learned, showing that the use of all annotated images (instead of only a few images near the annotated ones) improves the accuracy of the semantic segmentation.

Other methods try to find an alternative to the optical flow to avoid an increase in the processing cost. In [51], the temporal aspect is integrated into the form of additional constraints during learning, so as not to increase the inference cost. Another approach to integrating temporal information is to use Temporal Convolutions (TC) [52]. These convolutions are inspired by operations realized by neurons in Time Delay Neural Networks (TDNN) [53], in which samples of a signal feed different inputs of a neuron. Networks composed of layers of such temporal convolutions are called Temporal Convolution Networks (TCN). When applied to different sequential tasks, they have shown to get competitive results over RNN, LSTM and GRU units [54]. The temporal modules can be combined with any standard CNN to transform it into a spatio-temporal network. In [55], these temporal units are used after each layer of the encoder part of a Fully Convolutional Network (FCN). It is shown that the advantages of TC are that they can be added to existing architectures, and their ability to achieve almost the same accuracy as the ConvLSTM but with much fewer parameters. Space-time memory networks are another solution to leverage temporal redundancy in image sequences. Originally applied to object segmentation in videos [56], they have been further adapted to semantic segmentation in Temporal Memory Attention network [57].

#### 2.2.3. Need for Real-Time Processing

Improvements in models in terms of accuracy are often obtained at the expense of an increase in the processing cost. However, in the field of embedded systems like our application, the most important point is battery life. So, the performance criteria are inference time (or, alternatively, the number of floating point operations, which has the advantage to be independent of the hardware), and memory requirements (or, alternatively, the number of parameters of the CNN). Thus, accuracy is not the only performance criterion for a growing number of proposed models which try to get a better balance between accuracy and speed. Some other models are specifically thought to real-time processing. What is generally meant by real-time is a frame rate (FPS) greater than 30 on a single standard GPU. In [58], the optical flow is calculated in parallel with the segmentation, allowing real-time processing. In a Temporaly Distributed Network (TDNet) [59], temporal information is distributed on several subnets, each one processing an image of the input sequences. Another way of reducing the processing cost is to use skip convolutions in which convolutions are skipped in regions of images of low changes. In Dynamic Video Segmentation Network (DVSNet) [60], a segmentation network and a flow network are combined to get advantages of the two when used separately. GSVNet (Guided Spatially-Varying Convolution for Fast Semantic Segmentation) [61] uses lightweight flow estimation in 1/8-downscaled image space for temporal warping in segmentation output space. Fast Attention network (FANet) [62], which was mainly devoted to single image processing, has an extension to temporal processing. In [63], dynamic keyframe selection and distortion-aware feature rectification are used. In [64], an attention model captures the spatial consistency of low-level features in the temporal dimension. In [65], the DeepLabV3+ architecture is modified to get fast inference time on a GPU, and trained with a home-made dataset [7]. The system is then embedded on an EW for indoor and outdoor environments.

## 3. Description of Our Dataset and Test with a Semantic Segmentation Model

This section describes our contributions. The first one is the generation of a dataset of small sequences of virtual images representing outdoor scenes taken from viewpoints located on sidewalks. The second one consists of adapting an existing model to improve its accuracy and integrate the temporal dimension of the image sequences. A complementary test is a cross-validation between the two parts of our additional dataset, with the objective of quantifying the influence of image viewpoint in the quality of semantic segmentation.

### 3.1. Generation of a Virtual Dataset

#### 3.1.1. Overview

To generate our dataset, we have used CARLA simulator [1] (release 0.9.11), an open-source 3D simulator for experiments on autonomous vehicles, based on the Unreal Engine game engine. It comes with pre-made city environment maps for use. In the version that we have used, CARLA is distributed with 8 integrated maps as well as the following parameters:

- Addition of a varying density of pedestrians along the roads;
- Addition of vehicles on the roads (cars, bikes, motorcycles, vans, trucks, etc.);
- Variation of the weather conditions (amount of clouds, rain, puddles and wind);
- Variation of the time of day by modifying the position of the sun in the sky.

**Figure 1:**
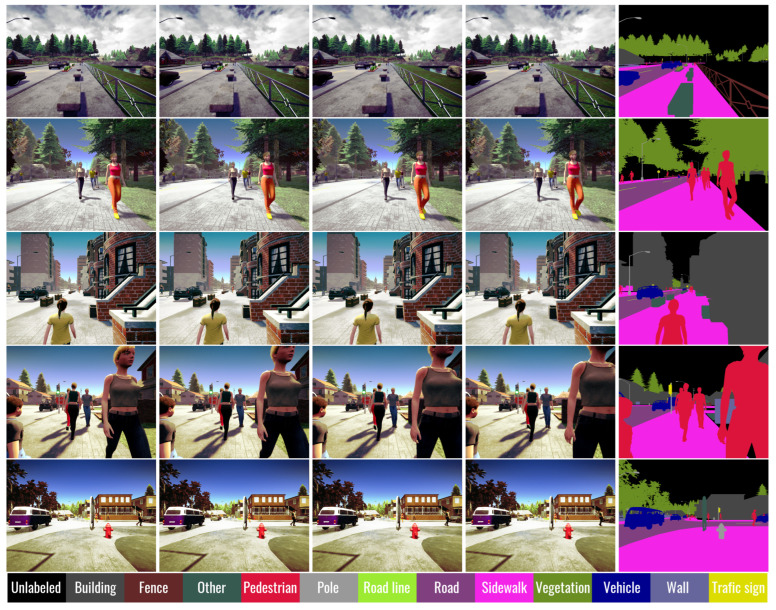
Examples of image sequences from the dataset. Each sequence is made of 4 images with a small gap between each. The column of images at the right is made of the ground-truth corresponding to the last RGB image of each sequence.

We have made use of these parameters to improve the diversity of situations in the images of the dataset, which is known to increase the generalization ability of a CNN model. Acquisition of image sequences was triggered at regular intervals when moving between “waypoints” in the virtual environment, and weather conditions were modified randomly between each capture. In the release of CARLA that we have used, semantic segmentation images are made up of 13 classes: Unlabeled, Building, Fence, Other, Pedestrian, Pole, Road line, Road, Sidewalk, Vegetation, Vehicle, Wall, and Traffic sign. The “Unlabeled” category corresponds to textures that are not part of an object, like sky or lawns (which are not part of “Vegetation”). In the “Other” category are found objects that are not included in the other classes like plant and flower pots. For our application, the most important categories are “Road” and “Sidewalk”, to find the way forward for an EW, as well as “Buildings” and “Poles” for obstacle avoidance. Figure 1 shows some example sequences of the dataset with the associated annotations for semantic segmentation.

We used 6 of the maps included in the simulator: Towns 1 to 5 and Town 7, with the maximum amount of vehicles available per map to simulate all possible scenarios (no traffic, low traffic, high traffic load, traffic jam). Sequences are made of 4 images taken with a small gap between each. The dataset is composed of 46,436 frames (11,609 sequences) partitioned in 41,024 frames (10,256 sequences) for train, 2696 frames (674 sequences) for validation, and 2716 for test (679 sequences). The size of the images is 800 × 600 (resp. width × height).

#### 3.1.2. Data Cleaning and Preprocessing

As the sequences of images are taken at regularly spaced times during the simulation of the movements of the vehicles, some of them are inconsistent (collision with objects, people, vegetation, etc). A manual cleaning process is thus needed to remove the corresponding sequences. After this cleaning process, around 12% of the images were deleted.

The only preprocessing that we have applied to the data is histogram equalization, in order to enhance the contrast within images.

#### 3.1.3. Additional Data

**Figure 2:**
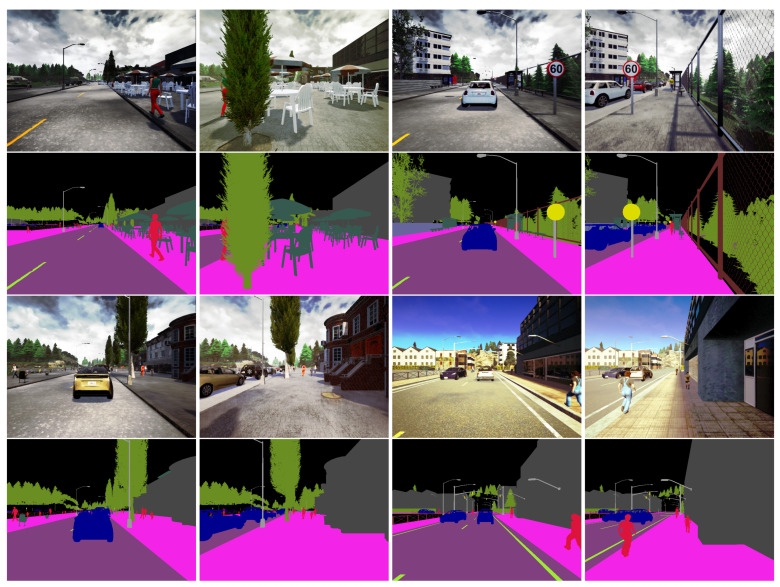
Four examples of couple-images from the additional dataset, taken from a viewpoint located on the road (left of each couple) and from a viewpoint located on the sidewalk (right of each couple). AT the couple of each couple-images, the true classes of the semantic segmentation are shown.

Additionally, we have generated another smaller dataset with images taken from 2 different viewpoints: one located on the road and the other located on the sidewalk. The number of frames of each part of this set (viewpoints on road/sidewalk) is 7288 (1822 sequences), partitioned in train/validation/test subsets. The numbers of frames of those subsets are, respectively, 5344 (1336 sequences), 500 (125 sequences) and 500. This smaller dataset is aimed at showing the importance of the viewpoint in the result of semantic segmentation. This can be done by cross-validation: learning on images taken from a viewpoint located on the road, and testing on images with a viewpoint located on the sidewalk, and vice versa. Figure 2 shows some sequences taken at the same position in the dataset, but from two different viewpoints (road/sidewalk).

### 3.2. Model Description

In order to do experiments with our dataset, we have designed a new convolutional neural network, based on DeepLabV3+ [2] architecture, but with additional techniques to adapt the model to image sequence processing and to improve performance of the baseline model in terms of accuracy.

#### 3.2.1. Architecture of the Network

In order to integrate temporal information from the inputs into the model, we have made use of Temporal Networks (TN) blocks, as proposed by [55] in Temporal Convolution Network (TCN). Within a block, the original TN has two layers of dilated convolutions with ReLU function, and residual connections [66], but for efficiency reasons, we use only one convolution layer. The final output of a TN block is the output of the convolutions added to the input of the block. Blocks also make use of pointwise 1D convolutions (of which kernel size is 1). The time dimension is processed as the convolution channel dimension and convolved over the flattened 1D vector CHW (Channels-Height-Width), to limit the increase of the processing cost.

For the encoder part of the network, we use ResNest blocks [67], which is a variant of ResNet blocks [66] where split-attention modules are used inside each block of ResNet. To integrate the temporal aspect of the data, we use temporal layers of the TCN type [55]. As the best place for temporal layers in the network is not known a priori, we have carried out different experiments, with three modes of operation: firstly, with a temporal layer between the encoder and the decoder, secondly, with additional temporal layers between each block of the encoder and thirdly, with both. The results of these experiments are given in the Section 4.

#### 3.2.2. Loss Function

The loss function is another means we have to try to improve the performance of the model in terms of accuracy. A survey of loss functions for semantic segmentation has been conducted in [68]. In our work, we have tested two loss functions: categorical cross-entropy and focal loss [69], which was initially proposed for object detection.

Categorical cross-entropy is the most popular loss function used is semantic segmentation tasks. It computes the difference between the one-hot ground-truth and the log softmax one of the network output. Categorical cross entropy is defined by Equation (1):

(1) $$Loss=-\sum_{i=1}^{n}y_{i}*\log{\hat{y}}_{i}$$

where *n* is the number of classes, $y_{i}$ the *i*th target value and ${\hat{y}}_{i}$ the *i*th output value (i.e., estimated value).

Focal loss is based on the principle of down-weighting easy examples and focusing on hard ones. A weight is given to each class of the segmentation depending on how high is the correct prediction rate for each class. It is defined as:

(2) $$FL=-\sum_{i=1}^{n}\alpha_{i}{\left(1-{\hat{P}}_{i}\right)}^{\gamma }\log{\hat{P}}_{i}$$

where *n* is the number of classes, $\alpha_{i}$ the static weight coefficient of the *i*th class, ${\hat{P}}_{i}$ the distribution of the prediction of that same class and γ a hyper-parameter to be tuned (γ > 0). In our experiments, we took γ = 2.

#### 3.2.3. Data Augmentation and Normalization

During the learning of the model, we have applied data augmentation, consisting of horizontal flipping with a probability of occurrence equal to 50%, and color jittering with 50% variation in brightness, contrast and saturation, in the same way for all images of a sequence. We have also added randomized affine transform (rotation on ±$15^{\circ }$, translation on ±15% of width and ±10% of height).

We have also applied pixel normalization (or “standardization”), consisting in applying computation to each RGB channel of the images to get zero mean and standard deviation equal to unity, over the whole dataset.

#### 3.2.4. Class Balancing

One of the main issues with training a network for semantic segmentation is a class imbalance where classes are not represented equally. If nothing is done to mitigate this issue, the network will be biased towards the most widely represented classes and will perform poorly on the rarest ones. We use class weighting to mitigate class imbalance by making the weights for the rare classes larger than with median frequency balancing, as in [70] (Equation (3)).

(3) $$w_{i}=1/\log\left(c+N_{i}/N\right)$$

with $w_{i}$ the weight for the *i*th class, $N_{i}$ the number of pixels of the *i*th class, *N* the total number of pixels and *c* (taken to 1.02) an additional hyperparameter allowing weights to be bounded in a given interval. Table 1 shows the weights values obtained for the different classes.

#### 3.2.5. Other Implementation Details

We have trained the network for 130 epochs among which the first 10 are used to warm up the SGD optimizer. We used a batch size of 4 and a time step of 4 when using video data. We used the SGD optimizer with the “poly” scheduler given by Equation (4):

(4) $$lr_{n}=lr\cdot 0.9^{n}$$

where lrn is the current learning rate, lr the initial one and *n* the current epoch. We used an initial learning rate of 0.0002, a momentum of 0.9 and a weight decay of 0.001. For efficiency reasons, our models are trained on 512 by 512 center-cropped images.

We used uniform label smoothing [71] to improve generalization of our model with a smoothing factor of 0.1.

## 4. Results

**Figure 3:**
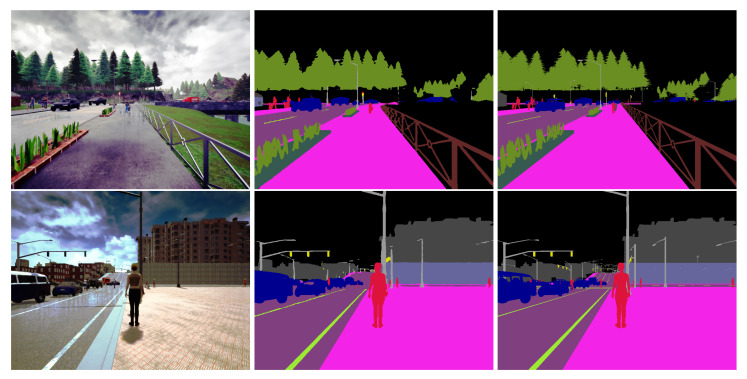
Two examples of model inference. From left to right: raw image, model prediction and ground truth.

As indicated above, we have tested three variants of our model: (1) without temporal layer; (2) with a temporal layer between encoder and decoder; (3) with temporal layers between each spatial layer of the encoder part and between encoder and decoder. We have also tested two different encoders: ResNet and ResNest. The best result was obtained for the third variant and ResNest encoder, with a mIoU (mean Intersection over Union) of 84.06% on the test set. Figure 3 shows some examples of inference of the model on images taken from the validation set.

Another test that we have made is cross-validation between the two parts of our additional dataset. The objective was to quantify the influence of point of view on the quality of semantic segmentation. For this test, we have used the non-temporal version of our model, as the objective is not to get the best accuracy as possible. Table 2 shows the result of this test. Those results show the importance of the viewpoint in a semantic segmentation task, confirming our motivation to develop a specific dataset for smart mobility on sidewalks.

## 5. Conclusions and Future Work

In this paper, we have presented a new dataset of small sequences of images dedicated to semantic segmentation tasks, generated with the virtual 3D environment CARLA. Images are taken from viewpoints located on sidewalks, to be useful for experiments on autonomous devices like EW. We also have presented experiments made with a CNN model adapted for temporal processing. Future work includes improving efficiency of the model that we tested, in terms of FPS, without sacrificing too much accuracy. Another important extension of this work is to develop a solution to domain adaptation from the virtual world to the real one, in view of the implementation of the model on the embedded system of a real wheelchair. Those two extensions would allow us to make real experiments. To go further, the issue of ground flatness cannot easily experiment with a simulator like CARLA, but the borders of sidewalks (e.g., step between road and sidewalk) could be detected by using the information of discontinuity in the depth map. In real experiments, specific irregularities or obstacles like holes or bumps in the ground could also be learned but would require a complementary dataset.

## Figures and Tables

**Table 1:** Our Carla 13 classes with their weights.

**Classes**	**Unlabeled**	**Building**	**Fence**	**Other**	**Pedestrian**	**Pole**	**Road Line**
Weights	8.7140	31.2160	30.7612	27.5623	27.9024	37.7695	8.1916
**Classes**	**Road**	**Sidewalk**	**Vegetation**	**Vehicle**	**Wall**	**Traffic Sign**	
Weights	4.9276	6.8403	33.7536	17.6963	46.7649	3.3284	-

**Table 2:** mIoU results of cross-validation test between the first part of the additional dataset: ADRoad (images taken from a viewpoint located on the road), and the second part: ADSidewalk (same images but taken from a viewpoint located on the sidewalk).

	Test on ADRoad	Test on ADSidewalk
learn on ADRoad	61.51%	30.58%
learn on ADSidewalk	51.32%	62.23%

## Data Availability

The dataset presented in this work is publically available at the following link: https://zenodo.org/record/6802655#.YuGrnRzP1Ea (accessed on 3 August 2022).

## Abbreviations

The following abbreviations are used in this manuscript:

ADAPT	Assistive Devices for disAbled People using robotic Technology
CNN	Convolutional Neural Networks
EW	Electric Wheelchair
FPS	Frames Per Second
mIoU	mean Intersection over Union
